# Complex Environment Path Planning for Unmanned Aerial Vehicles

**DOI:** 10.3390/s21155250

**Published:** 2021-08-03

**Authors:** Jing Zhang, Jiwu Li, Hongwei Yang, Xin Feng, Geng Sun

**Affiliations:** 1 College of Computer Science and Technology, Chang Chun University of Science and Technology, Changchun 130022, China; zhang_jing@cust.edu.cn (J.Z.); 2019100656@mails.cust.edu.cn (J.L.); fengxin@cust.edu.cn (X.F.); 2 Chongqing Research Institute of Changchun, University of Science and Technology, Chongqing 401122, China; 3 College of Computer Science and Technology, Jilin University, Changchun 130012, China; sungeng@jlu.edu.cn

**Keywords:** unmanned aerial vehicles, narrow passages, path planning, pruning, trajectory prediction

## Abstract

Flying safely in complex urban environments is a challenge for unmanned aerial vehicles because path planning in urban environments with many narrow passages and few dynamic flight obstacles is difficult. The path planning problem is decomposed into global path planning and local path adjustment in this paper. First, a branch-selected rapidly-exploring random tree (BS-RRT) algorithm is proposed to solve the global path planning problem in environments with narrow passages. A cyclic pruning algorithm is proposed to shorten the length of the planned path. Second, the GM(1,1) model is improved with optimized background value named RMGM(1,1) to predict the flight path of dynamic obstacles. Herein, the local path adjustment is made by analyzing the prediction results. BS-RRT demonstrated a faster convergence speed and higher stability in narrow passage environments when compared with RRT, RRT-Connect, P-RRT, 1-0 Bg-RRT, and RRT${}^{*}$. In addition, the path planned by BS-RRT through the use of the cyclic pruning algorithm was the shortest. The prediction error of RMGM(1,1) was compared with those of ECGM(1,1), PCGM(1,1), GM(1,1), MGM(1,1), and GDF. The trajectory predicted by RMGM(1,1) was closer to the actual trajectory. Finally, we use the two methods to realize path planning in urban environments.

## 1. Introduction

Unmanned aerial vehicles (UAVs) have gradually spread from military to civilian use and can be used in different areas, such as climate detection [1], environmental research [2], intelligent transportation [3], and rescue and search operations [4], by embedding various devices. For UAVs, performing missions in complex urban environments is more difficult than open space missions. Two main influencing factors are noted. First, path planning is difficult because the space is divided by buildings.

Second, the uncertainty of the trajectory of dynamic flight obstacles affects the safety of UAVs. Herein, path planning in a complex environment can be divided into global path generation and local path adjustment. The goal of global path planning is to plan the most efficient path in the shortest time in the overall static space, and local path adjustment is used to avoid threatening dynamic flight obstacles. Path planning for UAVs in complex environment is promising for future development.

Many narrow passages are found in urban environment, and multiple divided spaces may be connected by several narrow passages. Performing global path planning in narrow passages that have a smaller volume than the overall space is difficult. The calculation of path planning algorithm based on graph search is complicated [5,6,7]. The artificial potential field method [8] easily falls into local shocks and fails at path planning. Swarm intelligence [9,10] and neural networks [11,12] require long iterations or training. The sampling-based rapidly-exploring random tree (RRT) algorithm has shown outstanding performance.

RRT [13] was proposed for path planning with non-completion constraints of vehicle dynamics/kinematics. RRTs, which are efficient in solving complex spatial path solving problems, have no preprocessing. In addition, the RRT algorithm is simple to implement. For algorithms that are based on graph search, RRT calculations, which are also applicable to swarm intelligence and neural network algorithms, are simpler. However, the randomness of RRT enables the algorithm to generate more useless branch nodes, and they may spend considerable time in identifying and passing through unknown narrow passages.

Local path adjustment depends on the known trajectory information of dynamic flight obstacles. Therefore, a high-precision prediction model is a guarantee for UAVs to avoid dynamic obstacles. Regression analysis prediction model aims to make predictions by analyzing the functional relationship between observation data and statistical methods [14,15]. The autoregressive moving average model uses a mathematical model to approximately describe the data series generated by the object with the change in time [16,17]. The Markov prediction model uses the method of probability theory to study the change law of random events to predict the future state [18,19].

The neural network uses the steepest descent method to adjust the weights and thresholds through the back propagation of errors to predict the future trend [20,21]. Considering that more data will be processed, the time consumption when building the model is usually high. However, the grey prediction model [22] takes small data as the research object, and it has a relatively simple processing method for the original data. This model has high prediction accuracy. Related prediction models have been used in various aspects [23,24,25]. GM(1,1) is used as the representative model of the grey prediction model, which is especially suitable for this kind of forecasting. The contributions of this paper are presented as follows:

- In this paper, a BS-RRT algorithm is proposed to solve the global path planning problem. The algorithm converges quickly in the urban environment with narrow passages, and the algorithm has remarkable stability.
- To optimize the BS-RRT path further, a cyclic pruning algorithm is proposed to optimize the global programming path, and the effectiveness of the algorithm increases with the path’s tortuous degree.
- Local path adjustment depends on the prediction model. To improve the accuracy of the prediction model, this paper improves the calculation of the background value of the GM(1,1) model. At the same time, combined with the idea of metabolism, (the new method is presented to optimize the GM(1,1) model in this paper), which improves the prediction accuracy of the model greatly.
- This paper provides a feasible path planning scheme for UAV flight in a complex environment.

## 2. Related Work

Thus far, RRTs have been widely used to solve path planning problems in many fields. However, RRTs grow themselves incrementally by random sampling point. Meanwhile, in the case of narrow passages in urban space, the small volume of narrow passages leads to a lower probability of random sampling points being generated in the narrow passageway compared with the volume of the overall space, which costs more time to sample wide open areas before passing the narrow passage. One solution is to vary the randomness of the sampling points, which means that sampling points occur with different probabilities in various areas. According to the exploration results of the RRT algorithm in Yershova, A. [26], the sampling area of the sampling point is limited.

Jaillet, L. et al. [27] used adaptive sampling to adjust the sampling interval dynamically according to the current situation. In [28], the author adjusted the probability of random points appearing at the end point to guide the growth of RRT. The algorithm has high efficiency in the environment with a narrow passage, but the efficiency of the algorithm drops with the increase of the complexity of the environment. In [29], the algorithm grows in a favorable direction without collision by controlling the probabilistic diffusion and analyzing the results of the diffusion algorithm.

In [30], the author used the utility-oriented model to guide the random tree to allocate more computing resources in complex areas. Another approach is to generate multiple exploration trees simultaneously in space to deal with the narrow channel problem. In [31], the author proposed that two growing trees grow from the starting point and the end point. In [32], the author proposed an incremental path planning method that has obvious effect on solving local paths. In [33], the author proposed a parallel algorithm (C-FOREST) that is used to query the shortest path and grow multiple search trees simultaneously in parallel.

In [34], the authors extended the bidirectional tree to a multi-tree framework where any possible samples can be stored, and new trees are constructed. The aforementioned works have made some progress in RRT path planning. However, RRT expansion and connection is still a difficult problem for complex environments, and the time difference of each completion of path planning is large. Thus, the stability of the algorithm is low. The RRT algorithm reduces local re-sampling to a certain extent by changing the sampling strategy, which is prone to local oscillations before it passes through narrow passages, thereby, wasting certain computing resources.

In many cases, the whole space will be filled faster by the expansion of multiple trees, whereas the path search of narrow passage takes more sampling time. In this paper, BS-RRT is proposed to solve the problems of long time required to plan the global path of narrow passage; meanwhile, it has small time fluctuations in each planning. The initial RRT expansion is guided by a greedy mind to continuously expand toward the end point, which avoids wasting excessive computing resources in spacious space. Branch selection is conducted to pass the narrow passages when BS-RRT runs into obstacles. The optimal branch leaf node is selected as the root node to greed grow until connected to the end. Loop pruning is used to optimize the global path when connected from start to end.

When the global path is planned, the UAV needs to avoid local dynamic flight obstacles during the flight, thereby, changing the local path. The local path adjustment of UAV depends on the prediction results of the future trajectory of dynamic flight obstacles. the grey prediction model is suitable for predicting the trajectory of dynamic flight obstacles. The accuracy of background value estimation affects the accuracy of the model [35,36]. In [37], the author improved the accuracy and adaptability of the model by combining particle swarm optimization with the TWGM(1,1) algorithm to optimize the order and background value coefficient.

In [38], the GM (1,1) model was optimized using the Lagrange mean value theorem and interpolation coefficient method, and a background value that was correlated with the new variable K was constructed. Meanwhile, the accuracy of the model was improved by adding parameters. In [39], the author used an exponential curve, power function curve, polynomial curve, and interpolation function to optimize the background value, and the simulation results showed that these methods improved the accuracy of the model very well. In [40], the author improved the accuracy of the model by using piecewise cubic interpolation splines to reconstruct the background values while maintaining the monotonicity of the accumulated data.

In [41], the author used particle swarm optimization to optimize the development coefficient of the grey model, optimized the initial value of GM(1,1), and introduced the sliding window to improve the accuracy of the model. However, most models have low accuracy of long-term prediction performance because they do not consider the principle of new information priority. Swarm intelligence algorithm takes a long time to optimize the model. Runge phenomenon [42] may occur in the optimization method of high-order interpolation, which may affect the accuracy of the model.

In this paper, the GM(1,1) model is combined with the idea of metabolism, the principle of new information is fully considered priority, and the length selection of the model is discussed. At the same time, the 1-AGO dynamic sequence prediction model is combined with the Romberg quadrature formula to recalculate the background value to improve the prediction accuracy of the model. The results of the predictive model are used by the UAV as a reference to adjust the local path. In this paper, the global path planning algorithm is combined with the prediction model to realize the autonomous flight of UAV in complex environment.

The remainder of this paper is organized as follows. In Section 3, we explain the principle of the BS-RRT algorithm and cyclic pruning algorithm, the optimization method of RMGM(1,1), and the scheme of local path adjustment. In Section 4, we compare the performance of BS-RRT and the cyclic pruning algorithm with RRT, RRT-Connect, P-RRT, 1-0 Bg-RRT [28], and RRT${}^{*}$ through simulation experiments in a narrow passage, and we compare the prediction accuracy of RMGM(1,1) with ECGM(1,1), PCGM(1,1), GM(1,1), MGM(1,1), and GDF. Moreover, we verify the feasibility of the local path adjustment scheme. In Section 5, we present the summary and conclusions.

## 3. Principles of Path Planning in Complex Environment

The goal of path planning is to plan the most effective path in a short time. First, we briefly introduced the original RRT path planning algorithm. The principle of the improved algorithm BS-RRT and cyclic pruning algorithm for global path planning are described in detail in Section 3.1. Second, when the global path planning is finished, the UAV needs to predict the future trajectory of the captured dynamic flight obstacles in the process of flying along the trajectory.

We use the optimized RMGM(1,1) model to complete the prediction of the future flight trajectory of dynamic flight obstacles. The modeling process and error analysis of GM(1,1) and the modeling principle of RMGM(1,1) are introduced in Section 3.2. Finally, in Section 3.3, we describe a local path adjustment scheme that makes the path adjustment according to the predicted results of Section 3.2. We use a combination of global path planning and a local path adjustment scheme to achieve autonomous flight of the UAV.

### 3.1. Global Path Planning

#### 3.1.1. Principle of RRT

**Figure 1:**
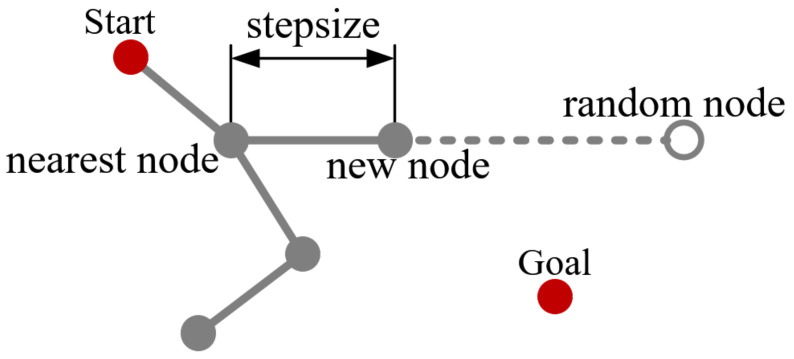
Principle of RRT extension.

The RRT constructs a tree-like data structure to incrementally explore unknown space by randomly sampling points. Figure 1 shows the schematic of the RRT extension. First, a random node is generated with random coordinate in space. Second, the tree is traversed by RRT to find the node closest to the sampling point. Third, a new node of one step along the direction that this node points to the sampling point. Finally, whether collision occurs from the nearest node to the new node is verified. Collision validation determines whether new nodes can be inserted into the tree. The above process is repeated until the algorithm finds a path that connects the starting point and the point goal.

#### 3.1.2. Principle of BS-RRT

The expansion process of BS-RRT is divided into three parts. First, the expanded tree grows greedily along the direction of the end point. Second, when the expanding tree encounters an obstacle, it branches and grows to pass the obstacle. Finally, after the expanded tree passes the obstacle, the leaf node of the branch with the closest Euclidean distance to the end point among the branches is selected to grow again greedily. These three steps are circulated, in turn, to finally generate a path connecting the starting point and the goal point.

**Figure 2:**
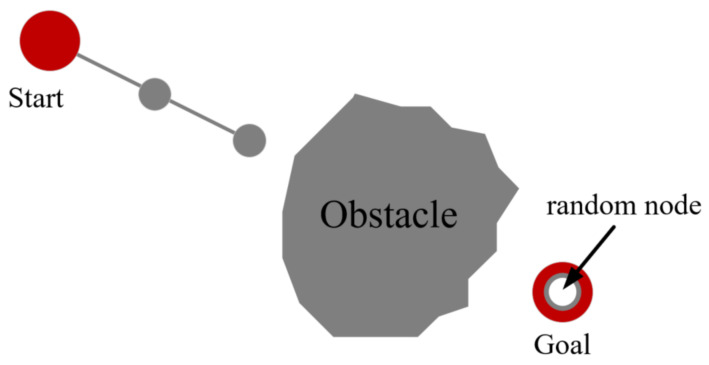
Greedy growth.

**Figure 3:**
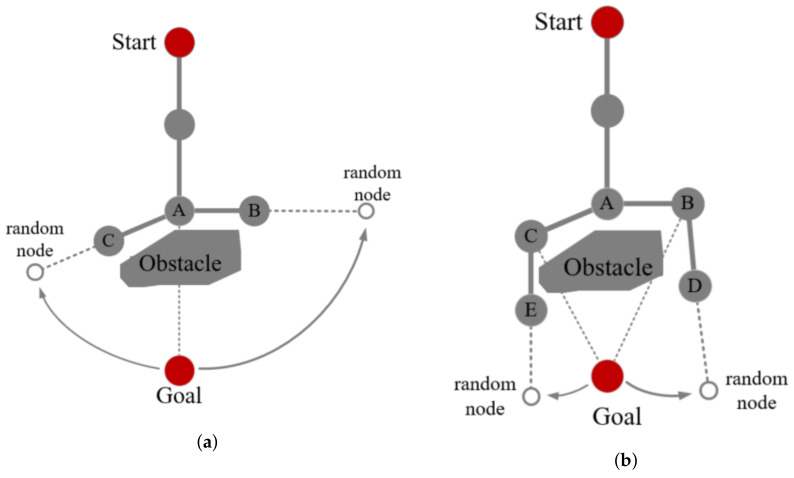
(**a**) shows the first branch growth. The point A is the root node, the point C and the point B are the leaf nodes; (**b**) shows the second branch growth. The point C and the point B are the root nodes, the point E and the point D are the leaf nodes.

- Greedy growth
  In the initial phase, the sampling strategy is changed to speed up the tree expansion, and the sampling point for each sampling coincides with the goal point. In Figure 2, the BS-RRT continues to expand toward the goal point. The algorithm goes to the next stage when the BS-RRT fails to grow.
- Branch growth
  When the greedy growth stops, the BS-RRT tree will enter the branch growth stage to avoid obstacles. Point *A* in Figure 3a represents that point *A* failed to extend the next greedy point, and thus the point enters the branch, and point *A* is set as the root point of the branch. The line from the root point to the goal point divides the space into left and right regions. In the left region, the sampling point gradually moves away from the goal point in a clockwise trend each time, and the sampling stops until point *C* is expanded successfully. The sampling point in the right region gradually moves away from the goal point counterclockwise each sampling, the sampling stops until point *B* is extended successfully. In Figure 3b, points *B* and *C* cannot grow greedily because obstacles are observed in the direction from point *B* and *C* to the goal point. Hence, they continue to branch.
  The sampling point from point *C* to the direction of the goal point moves clockwise away from the goal point for each sampling until point *E* is expanded successfully. Similarly, the sampling point from point *B* points to the direction of the goal point moves counterclockwise away from the goal point each sampling until point *D* is expanded successfully. At this point, points *E* and *D* can grow greedily, and branch growth ends. Notably, in the branching stage, the length of the extension of all nodes is equal to the initial step size.
  Other branch nodes only expand one child node in addition to the original root point *A*. In the regions divided by root point *A*, the sampling point will only shift the sampling clockwise in the left region or counterclockwise sampling on the right, such as points C and E expand clockwise, meanwhile, points B and D expand counterclockwise. The branch growth in the corresponding region deviated to the direction of the goal point pointing to the leaf point to stop until the sampling point.
- Branch selection
  When the branches stop growing, this indicates that BS-RRT has passed the barrier and enters the branch selection stage. If one branch fails to grow, then another branch will be selected directly. The overall BS-RRT algorithm will fail when both branches fail. The algorithm selects the branch with a short Euclidean distance from the goal point when the branches grow successfully. The selected leaf point of the branch as the starting point goes to the first step of greedy growth.

#### 3.1.3. Cyclic Pruning Algorithm

The aim of cyclic pruning algorithm is to optimize the path generated by BS-RRT, and the shortest path from the start point to the end point will be obtained accordingly. The generated path will be pruned twice by a cyclic pruning algorithm. Each point in the path from the start point to the end point is denoted from 1 to *n*. The first pruning will significantly reduce the number of unnecessary nodes as well as the path length, and the second pruning will shorten the path length while keeping the number of nodes unchanged.

**Figure 4:**
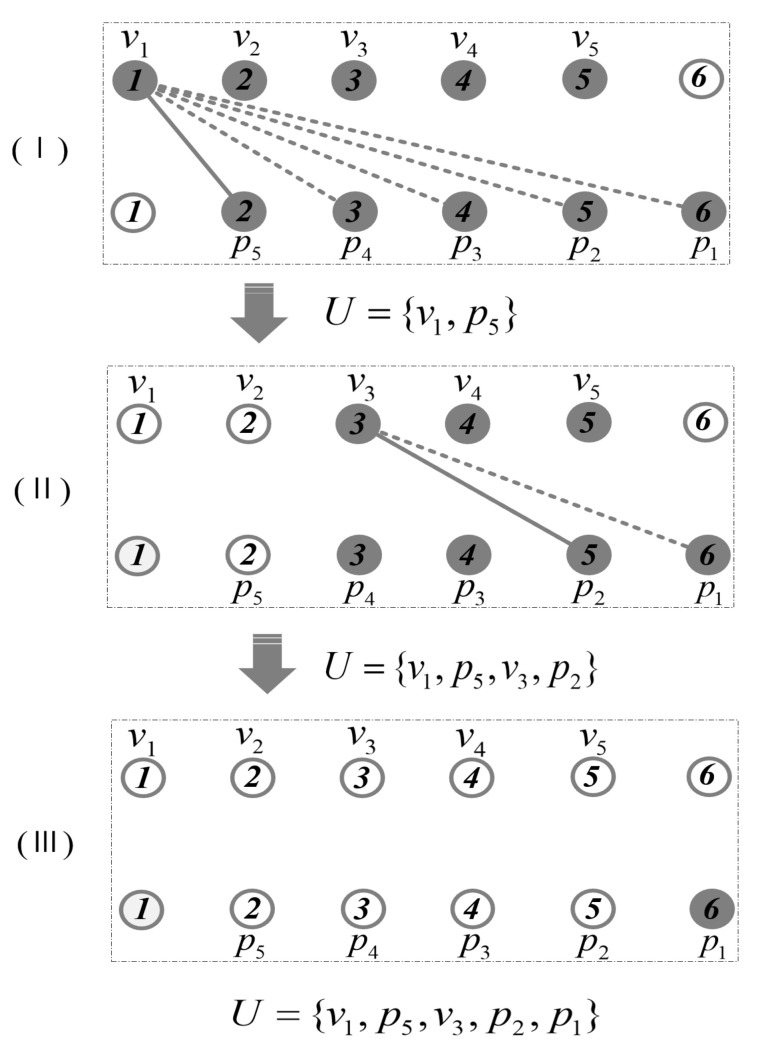
First pruning of the path, (**I**) shows the point *v*_1_ and the point *p*_5_ are connected successfully; (**II**) shows the point *v*_3_ and the point *p*_2_ are connected successfully; (**III**) shows no point can connect successfully.

**Figure 5:**
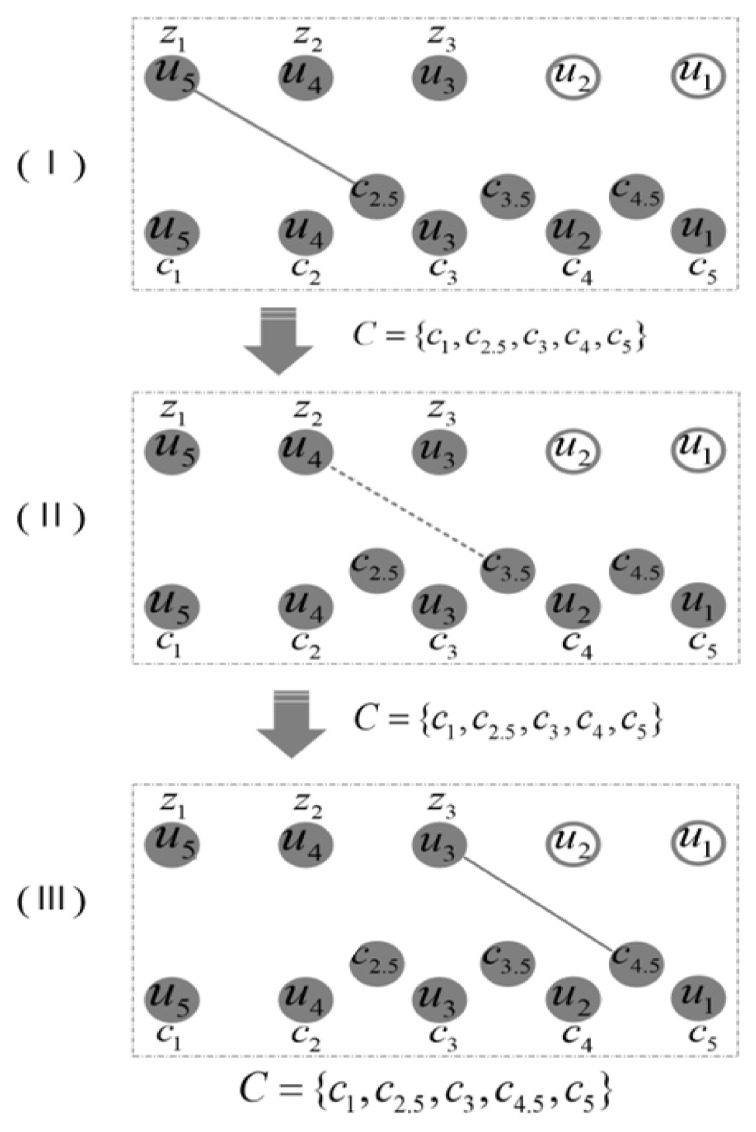
Second pruning of the path, (**I**) shows the point $z_{1}$ and the point $c_{2.5}$ are connected successfully; (**II**) shows the point $z_{2}$ and the point $c_{3.5}$ fail to connect; (**III**) shows the point $z_{3}$ and the point $c_{4.5}$ are connected successfully.

- The first pruning
  We define the following set: set *V* = {$v_{1}$, $v_{2}$,$v_{3}$,…, $v_{n-1}$}, set *P* = {$p_{1}$, $p_{2}$, $p_{3}$,…, $p_{n-1}$}, and empty set *U*. Set *V* traverses every point in the path from the start point to the n−1th point, set *P* traverses each point in the path from the end point *n*th to the third point in reverse, and the point in the set records the coordinate information of every point in the path. Based on the above definition, the point connecting to itself will be avoided.
  The first pruning of the path is performed as follows. First, we determine whether the point $v_{i}$ in the set *V* is connected to any point in set *P*. If the point $v_{i}$ and the point $p_{j}$ can be connected with no obstacle, then the points from $v_{i}$ to $v_{n-j+1}$ are deleted from the set *V*, the points from $p_{n-i+1}$ to $p_{j}$ are deleted from the set *P* as well. Meanwhile, the points $v_{i}$ and $p_{j}$ are added to set *U*.
  Repeat the above process until the set *V* is empty, point $p_{1}$ will be added to the set *U* if the point $p_{1}$ is not in the set *U*. The set *U* is the final result set after the first pruning. An example is shown in Figure 4 to describe the process of the first pruning in detail. There are seven points in the path generated by BS-RRT, where the hollow circles represent points that are not in sets *V* and *P*, dashed lines represent two points fail to connect due to the existence of obstacles, and solid lines represent two points can be connected directly. Thus, set *V* = {$v_{1}$,$v_{2}$,$v_{3}$,$v_{4}$,$v_{5}$} and set *P* = {$p_{1}$, $p_{2}$, $p_{3}$,$p_{4}$, $p_{5}$} can be constructed. The first pruning operation is divided into three-steps in Figure 4.
  First, in determining whether the point $p_{j}$ in the set *P* can be connected to point $v_{1}$, the initial value of *j*, *j* is set as 1. Figure 4I shows that point $v_{1}$ and $p_{5}$ can be connected directly. Then, points $v_{1}$ and $v_{2}$ are deleted from set *V*, point $p_{5}$ is deleted from set *P*, and point $v_{1}$ and $p_{5}$ are added to set *U*.
  Second, we determine whether the point $p_{j}$ in the set *P* can be connected to point $v_{3}$ in turn, and point $v_{3}$ and $p_{2}$ can be connected directly as shown in Figure 4II. Points $v_{3}$, $v_{4}$, and $v_{5}$ are deleted from set *V*, points $p_{2}$, $p_{3}$, and $p_{4}$ are deleted from set *P*, and points $v_{3}$ and $p_{2}$ are added to set *U*.
  Finally, the set *V* is empty in Figure 4III, the point $p_{1}$ will be added to the set *U* if $p_{1}$ is not in the set *U*. Thus, the set *U* = {$v_{1}$,$p_{5}$,$v_{3}$,$p_{2}$,$p_{1}$}. All of points in the set *V* can be connected in turn, and the first pruning is finished.
- The second pruning
  Assume that there are m points in the set *U* after the first pruning, we define the following sets, set *C* = {$c_{1}$,$c_{2}$,$c_{3}$,…, $c_{m}$}, set *Z* = {$z_{1}$,$z_{2}$,$z_{3}$,…, $z_{m-2}$}. The points in set C represent the points in set *U* starting from $u_{m}$ to $u_{1}$. The points in set *Z* represent the points in set *U* starting from point $u_{m}$ to point $u_{3}$. The missing points $u_{1}$ and $u_{2}$ in set *Z* ensures that the connection of the last two points is not on the same line segment.
  The second pruning operation is performed as follows. Determining whether the first point $z_{1}$ in the set *Z* can be connected to the midpoint of the two points in the set *C*, that is, the midpoint of $c_{i}$ and $c_{i+1}$ connecting $z_{i-1}$. If the connection is successful, then the midpoint of $c_{i}$ and $c_{i+1}$—namely node $c_{i+0.5}$—will replace the point $c_{i}$ in set *C*. Point $z_{i+1}$ will be determined in turn. After all the points in the point set *Z* are tested, the points in the set *C* forms the final path, and the cyclic pruning algorithm ends.
  An example is shown in Figure 5 to describe the process of the second pruning in detail. There are five points in the set *U*, the hollow circles represent points that are not in sets *Z*, dashed lines represent the failed connection between two points, and solid lines represent successful connections between two points. Set *C* = {$c_{1}$,$c_{2}$,$c_{3}$,$c_{4}$,$c_{5}$} and set *Z* = {$z_{1}$,$z_{2}$,$z_{3}$} are constructed accordingly.
  Similarly, the second pruning operation is divided into three steps in Figure 5. First, node $z_{1}$ is connected to node $c_{2.5}$ successfully, and then point $c_{2.5}$ is added to the set *C* to replace point $c_{2}$. Second, point $c_{3.5}$ will not add to the set *C* because point $z_{2}$ failed to connect to point $c_{3.5}$. Finally, point $c_{4.5}$ will replace point $c_{4}$ in the set C because point $z_{3}$ can be connected to node $c_{4.5}$ with no obstacle. The cyclic pruning algorithm ends and the set *C* is the final pruning result.

### 3.2. Trajectory Prediction of Dynamic Obstacles

We assume that the coordinate sequence of a dynamic obstacle is P(k)=(x(k),y(k),z(k)), k=1,2,…,n. x(k),y(k) and z(k) are coordinates for the X,Y, and *Z* axes at time *k*. The map is constructed in the first quadrant to make the values of the coordinate sequence non-negative. Three sets of data sequences (x(1),x(2),…,x(n)), (y(1),y(2),…,y(n)), and (z(1),z(2),…,z(n)) are obtained by splitting the original coordinate sequence P(k). Three predicted values $\hat{x}\left(n+1\right)$, $\hat{y}\left(n+1\right)$, and $\hat{z}\left(n+1\right)$ are obtained by building a prediction model for the three sets data series respectively, and the predicted coordinates $\hat{p}\left(n+1\right)=(\hat{x}\left(n+1\right),\hat{y}\left(n+1\right),\hat{z}\left(n+1\right)$ are obtained.

In this part, the X axis coordinate sequence (x(1),x(2),…,x(n)) is used as an example to demonstrate the main modeling steps of the GM(1,1) model, and then the error of the model is analyzed. We combine the first-order cumulative dynamic sequence prediction model and Romberg’s numerical integration formula to recalculate the background value to reduce the error of the model. We also rebuild the model with the idea of metabolism.

#### 3.2.1. Principle and Error Analysis of GM(1,1) Modeling

**Figure 6:**
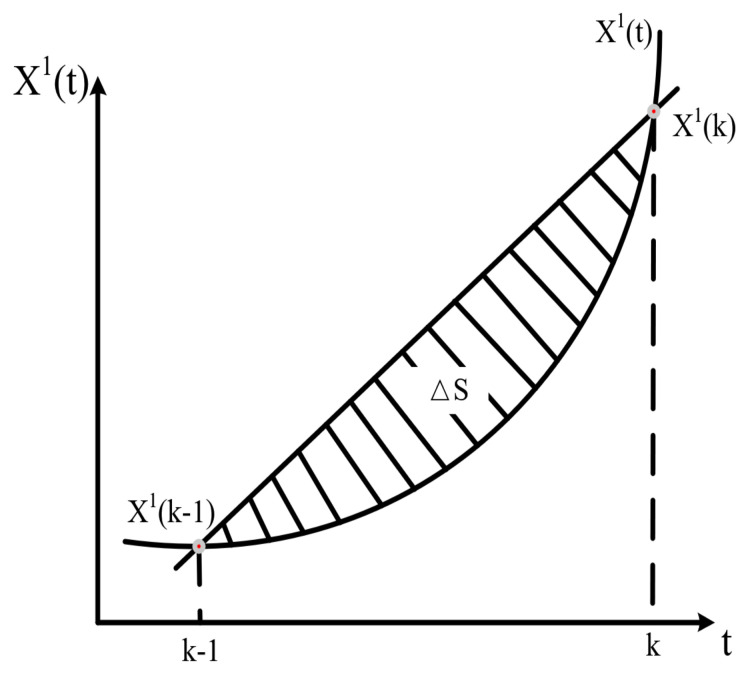
Reasons for GM(1,1) model error.

- Modeling of Grey Model GM(1,1)
  Let the original non-negative sequence data be
  (1) $$X^{\left(0\right)}=\left(x^{\left(0\right)}\left(1\right),x^{\left(0\right)}\left(2\right),\ldots ,x^{\left(0\right)}\left(n\right)\right).$$
  From Equation (1), $x^{\left(0\right)}\left(k\right)=x\left(k\right)$, and digital (0) indicates that the initial data sequence. The $X^{\left(1\right)}$ is given as follows
  (2) $$X^{\left(1\right)}=\left\{x^{\left(1\right)}\left(1\right),x^{\left(1\right)}\left(2\right),\ldots ,x^{\left(1\right)}\left(n\right)\right\}.$$
  where
  (3) $$x^{\left(1\right)}\left(k\right)=\sum_{i=0}^{k}x^{\left(0\right)}\left(k\right)=x^{\left(1\right)}\left(k-1\right)+x^{\left(0\right)}\left(k\right),k=1,2,\cdots ,n.$$
  Equation (3) is first-order accumulated generation operating (1-AGO) series of $X^{\left(0\right)}$. From Equation (3), we suppose that sequence $X^{\left(1\right)}$ meets the following first-order grad forecasting differential equation
  (4) $$\frac{dx^{\left(1\right)}\left(t\right)}{dt}+ax^{\left(1\right)}\left(t\right)=b.$$
  The solution of Equation (4) with the initial condition ${\hat{x}}^{\left(1\right)}\left(1\right)=x^{\left(1\right)}\left(1\right)$ is presented as follows
  (5) $${\hat{x}}^{\left(1\right)}\left(k+1\right)=\left(x^{\left(0\right)}\left(1\right)-\frac{b}{a}\right)e^{-ak}+\frac{b}{a},k=1,2,3,\ldots ,n.$$
  Thus, we obtained the following grey prediction equation
  (6) $$\begin{array}{cc} {\hat{x}}^{\left(0\right)}\left(k+1\right) & ={\hat{x}}^{\left(1\right)}\left(k+1\right)-{\hat{x}}^{\left(1\right)}\left(k\right)\\ \; & =\left(1-e^{a}\right)\left(x^{\left(0\right)}\left(1\right)-\frac{b}{a}\right)e^{-ak},k=1,2,3,\ldots ,n. \end{array}$$
  Here, k=n, ${\hat{x}}^{\left(1\right)}\left(n+1\right)$ is the predicted coordinate on the X axis. To obtain the prediction model of the raw data sequence, we need to determine the grey development coefficient *a* and the grey control parameter *b* in Equation (4). For this purpose, we performed the integral accumulation on both sides of Equation (4) for every contiguous interval, and then we can obtain
  (7) $$x^{\left(1\right)}\left(k+1\right)+a\int_{k}^{k+1}x^{\left(1\right)}\left(t\right)dt=b.$$
  Let the background value be
  (8) $$z^{\left(1\right)}\left(k+1\right)=\int_{k}^{k+1}x^{\left(1\right)}\left(t\right)dt.$$
  Consequently, to estimate the values of *a* and *b*, we must use some methods to estimate the background value $z^{\left(1\right)}\left(k+1\right)$. We yield the estimated background value $z^{\left(1\right)}\left(k+1\right)$ as follows
  (9) $$z^{\left(1\right)}\left(k+1\right)=\frac{1}{2}\left[x^{\left(1\right)}\left(k\right)+x^{\left(1\right)}\left(k+1\right)\right].$$
  The method of solving a,b parameters is expressed as
  (10) $$\hat{\alpha }={\left(a,b\right)}^{T}={\left(B^{T}B\right)}^{-1}B^{T}Y,$$
  (11) $$B=\left[\begin{array}{cc} -z^{\left(1\right)}\left(2\right) & 1\\ -z^{\left(1\right)}\left(3\right) & 1\\ \vdots & \vdots \\ -z^{\left(1\right)}\left(n\right) & 1 \end{array}\right],Y=\left[\begin{array}{c} x^{\left(0\right)}\left(2\right)\\ x^{\left(0\right)}\left(3\right)\\ \vdots \\ x^{\left(0\right)}\left(n\right) \end{array}\right].$$
- Error analysis
  Equation (8) shows that the classical GM(1,1) model uses the average of adjacent values to estimate the background value $z^{\left(1\right)}\left(k\right)$. Its geometric meaning is that the trapezoidal area, which is based on the edge of exponential curve $x^{\left(1\right)}\left(t\right)$, has been replaced by the area of straight ladder. This method has significant error when the 1-AGO data sequence varies greatly. As shown in Figure 6, ΔS is the error existing in the model.
  According to the error analysis results, the curve $x^{\left(1\right)}\left(t\right)$ has fitting by first-order accumulation (1-AGO) dynamic sequence prediction model [43], and the trapezoidal area, which is based on the edge of the exponential curve $x^{\left(1\right)}\left(t\right)$, was recalculated by the Romberg numerical integration formula. Meanwhile, the optimized model was constructed by combining the idea of metabolism, named RMGM(1,1).

#### 3.2.2. Principle of RMGM(1,1) Modeling

In this part, the X axis coordinate sequence (x(1),x(2),…,x(n)) is used as an example in the same way to describe the principles of the 1-AGO dynamic sequence prediction model briefly and Romberg’s numerical integration formula, as well as the construction of the RMGM(1,1) model.

- 1-AGO dynamic sequence prediction model [43]
  Assume that Equations (1) and (2) meet the following law: If $x^{\left(0\right)}\left(k\right)$ has the form of homogeneous exponential growth $x^{\left(0\right)}\left(k\right)$=$ce^{a(k-1)}$, then the 1-AGO sequence is in the form of non-homogeneous exponential sequence (i.e., Equation (12)) and vice versa.
  (12) $$x^{\left(1\right)}\left(k\right)=Ae^{\alpha (k-1)}+B,k=1,2,\cdots ,n.$$
  The solving formula of each parameter is expressed as follows
  (13) $$\alpha =\ln\frac{x^{\left(0\right)}\left(k\right)}{x^{\left(0\right)}\left(k-1\right)},$$
  (14) $$A=\frac{x^{\left(0\right)}\left(k\right)}{{\left(\frac{x^{\left(0\right)}\left(k\right)}{x^{\left(0\right)}\left(k-1\right)}\right)}^{k-1}\left(1-\frac{x^{\left(0\right)}\left(k-1\right)}{x^{\left(0\right)}\left(k\right)}\right)},$$
  (15) $$B=x^{\left(0\right)}\left(k_{1}\right)-A=x^{\left(0\right)}\left(k_{1}\right)-\frac{x^{\left(0\right)}\left(k\right)}{{\left(\frac{x^{\left(0\right)}\left(k\right)}{x^{\left(0\right)}\left(k-1\right)}\right)}^{k-1}\left(1-\frac{x^{\left(0\right)}\left(k-1\right)}{x^{\left(0\right)}\left(k\right)}\right)}.$$
  Equation (12) is called the 1-AGO dynamic sequence prediction model.
- Romberg quadrature formula
  The Romberg quadrature formula is also called the successively divided and semi-accelerated method. As an extrapolation algorithm, this formula improves the accuracy of the result of integration without increasing the amount of calculation. The Romberg quadrature formula has a more accurate integral approximate value by the weighted average of the approximate values of the trapezoidal formula.
  The initial parameter is set at k=1, and the integral is calculated roughly as
  (16) $$r_{1,1}=\frac{b-a}{2}\left[f\left(a\right)+f\left(b\right)\right].$$
  The value of the next position is calculated as
  (17) $$r_{k+1,1}=\frac{1}{2}\left[r_{k,1}+\frac{b-a}{2^{k-1}}\sum_{n=1}^{M}f\left(a+\left(2n-1\right)\frac{b-a}{2^{k}}\right)\right],M=2^{k-1}.$$
  A recursive formula is used for the calculation
  (18) $$r_{k+1,m}=\frac{4^{m-1}r_{k+1,m-1}-r_{k,m-1}}{4^{m-1}-1}.$$
  If the difference between $r_{k+1,k+1}$ and $r_{k,k}$ meets the predetermined accuracy ε, then the calculation stops. Otherwise, *k* goes up by 1, and then we proceed to Equation (17).
  (19) $$R=\left[\begin{array}{cccccc} r_{1,1} & & & & & \\ r_{2,1} & r_{2,2} & & & & \\ r_{3,1} & r_{3,2} & r_{3,3} & & & \\ r_{4,1} & r_{4,2} & r_{4,3} & r_{4,4} & & \\ r_{5,1} & r_{5,2} & r_{5,3} & r_{5,4} & r_{5,5} & \\ \vdots & \vdots & \vdots & \vdots & \vdots & \ddots \end{array}\right].$$
  The Romberg algorithm is expressed as the lower triangular matrix of Equation (18), and it can be extended infinitely downward and backward. The optimal approximate solution for a definite integral is $r_{k,k}$, which is the lower right item calculated in Equation (19). The calculation of the Romberg quadrature formula recurs in Equations (17) and (18).
- Building a metabolic model
  In the traditional modeling process, the prediction accuracy of the model is reduced continuously as the system status changes by selecting a fixed length of the original data to build a model. Therefore, the higher prediction accuracy of the model should be ensured by updating the modeling data. This process of building a model is dynamic. The new data will be added to the modeling data sequence, and the oldest data in the modeling data will be deleted to reconstruct the model when a new data appears, the length of the modeling data of the construction of model remains unchanged. The optimal length of the modeling data will be discussed in the experiment.
- Construction of RMGM (1,1) model
  The RMGM(1,1) model reduces error by calculating the area of curve of the 1-AGO dynamic sequence model in each interval by using the Romberg quadrature formula.
  **Step 1**: Select a fixed-length raw data sequence $x^{\left(0\right)}\left(k\right)$ and calculate 1-AGO sequence $x^{\left(1\right)}\left(k\right)$, k=1,2,3,…,n.
  **Step 2**: Let a=1,b=2, locate the interval [a,b], combine Equations (13)–(15) in this interval, and calculate the required parameters α, *A*, and *B* of Equation (12) to obtain the formula $f\left(k\right)=x^{\left(1\right)}\left(k\right)=Ae^{\alpha (k-1)}+B$.
  **Step 3**: Calculate *r* of the integral area of the interval [a,b] in accordance with Equations (16)–(18). The ordinate value of the background value curve required by Equations (16)–(18) is given by Step 2, Let $z^{\left(1\right)}\left(k\right)=r$.
  **Step 4**: if b<n, then proceed to Step 2, and let *a* and *b* increase by 1. The sequence of the background values $z^{\left(1\right)}\left(k\right)$ is obtained until b=n.
  **Step 5**: The time response function is obtained by combining Equations (7)–(10).
  **Step 6**: Newly generated data are added to the original data sequence $x^{\left(0\right)}\left(k\right)$, and the first old data are deleted at the same time. A new data sequence $x^{\left(0\right)}\left(k\right)$ is generated by keeping the length unchanged, and then return to Step 1.

The predicted value $\hat{x}\left(n+1\right)$, $\hat{y}\left(n+1\right)$, and $\hat{z}\left(n+1\right)$ is obtained by establishing the RMGM(1,1) model in three coordinate series (x(1),x(2),…,x(n)), (y(1),y(2),…,y(n)), and (z(1),z(2),…,z(n)). The prediction coordinates $\hat{p}\left(n+1\right)=(\hat{x}\left(n+1\right)$,$\hat{y}\left(n+1\right),\hat{z}\left(n+1\right)$ are obtained accordingly.

### 3.3. Local Path Adjustment

The local path of the UAV is adjusted according to the prediction results of the dynamic flight obstacle in the previous step. Path alignment schemes are divided into two approaches. First, the UAV tries to keep to the original planned flight path when it encounters threats, that is, the UAV only changes its speed. At this point, the path of the dynamic obstacle is not coincident from that of the UAV, and they will collide at the intersection point. The UAV makes a local deviation to avoid dynamic obstacles when the deceleration strategy is not feasible. This means that the path of the UAV and dynamic obstacle are coincident, and many points are closer and threatening on both of the paths. The UAV returns to the original path when it eludes the dynamic obstacles successfully.

**Figure 7:**
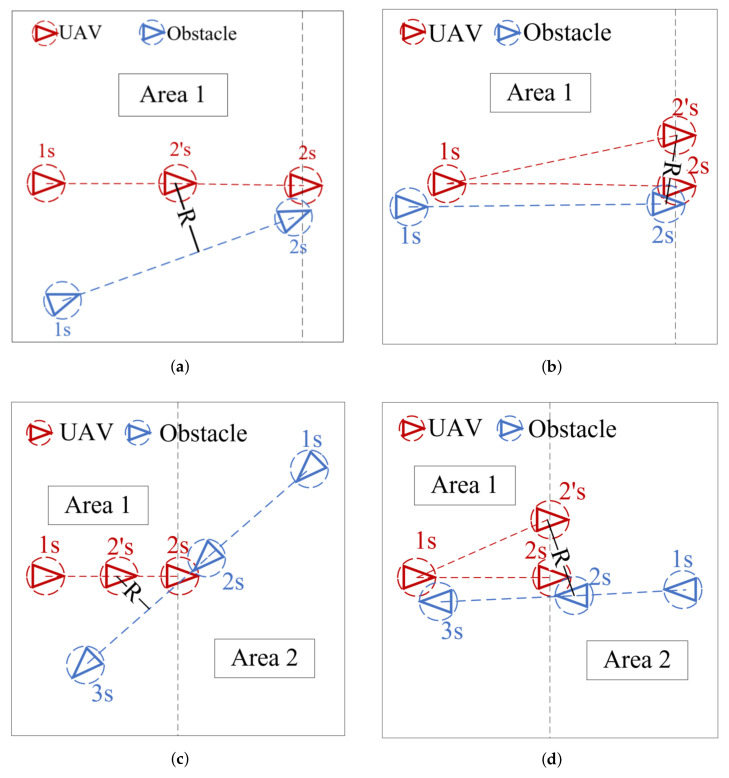
*R* refers to the distance recorded between the UAV and the obstacle in every second. (**a**,**b**) show the obstacle avoidance of UAV when the obstacle is co-directional; (**c**,**d**) show the obstacle avoidance of UAV when the obstacle is contralateral.

In Figure 7, the space is divided into two regions by making vertical lines perpendicular to the trajectory of the UAV according to the collision position. The dynamic flight obstacle in Region 1 is called the co-directional obstacle, and the dynamic flight obstacle in Region 2 is called the contralateral obstacle. The coordinates of 1 s indicate where the UAV has arrived in the path, and the coordinates of 2 s in the path represent where the UAV is going to be. According to the results of the prediction model, the UAV will collide with the dynamic flight obstacle at the position of 2 s. The coordinate of 2’s is added to the path instead of the coordinate of 2 s after the local path is adjusted. According to the different flight trajectories of dynamic flight obstacles, we analyze four collision situations and suggest a local path adjustment strategy for each collision.

- In Figure 7a, the UAV verifies whether the adoption of a deceleration strategy is feasible, that is, the coordinate of 2’s is added to the original path instead of the coordinate of 2 s. *R* refers to the distance recorded between the UAV and the obstacle in every second. *R* should be greater than the sum of the radius of the envelopment circle of UAV and dynamic flight obstacle if no collision occurs. The path of the codirectional obstacle is not coincident with the path of the UAV and the *R* value is far greater than the sum of the radius of the envelope circle of UAV and the dynamic flight obstacle at 2’s. Therefore, the UAV will adopt this strategy wherein the position at 2’s replaces the position at 2 s.
- In Figure 7b, the path of the dynamic obstacle is coincident of the UAV. If the deceleration strategy is adopted, then the *R* value is less than the sum of the radius of the envelope circle of the UAV and the dynamic flight obstacle, and the collision will still occur. A decentralized strategy is adopted, the position at the 2 s is offset at the 2’s while keeping *R* greater than the sum of the radius of the envelope circle of the UAV and the dynamic flight obstacle. The UAV returns to the original path at 3 s after avoiding the dynamic obstacle. For the opposite dynamic obstacle, verifying whether the deceleration strategy is feasible first is necessary.
- In Figure 7c, if the deceleration strategy is adopted, then *R* is greater than the sum of the radius of the UAV and the dynamic flight obstacle envelope circle at the 2’s, and the position at the 2’s instead of the position at the 2 s add the path. Hence, obstacle avoidance is successful.
- In Figure 7d, the path of the UAV and dynamic obstacle are coincident, and *R* will be less than the sum of the radius of the enveloping circle of UAV and dynamic flight obstacle if the deceleration strategy is adopted. Thus, the decentralized strategy is carried out. The position at the 2’s is the offset position, thereby, making the *R* value greater than the sum of the radius of the envelopment circle of the UAV and the dynamic flight obstacle. The UAV returns to the original path at 3 s after successful obstacle avoidance.

## 4. Experiments and Discussions

In this section, simulation experiments are carried out on an IntelCPU2.8G 16 GB memory computer using a MATLAB-R2016B environment. UAVs are low-energy IoT devices, which can save energy through effective energy management methods [44,45]. Energy saving can also be achieved by reducing unnecessary maneuver of UAVs. The three dimensional space is transformed into two dimensional plane to avoid the change of UAVs in vertical direction [46] in this paper. This section is divided into three parts.

**Figure 8:**
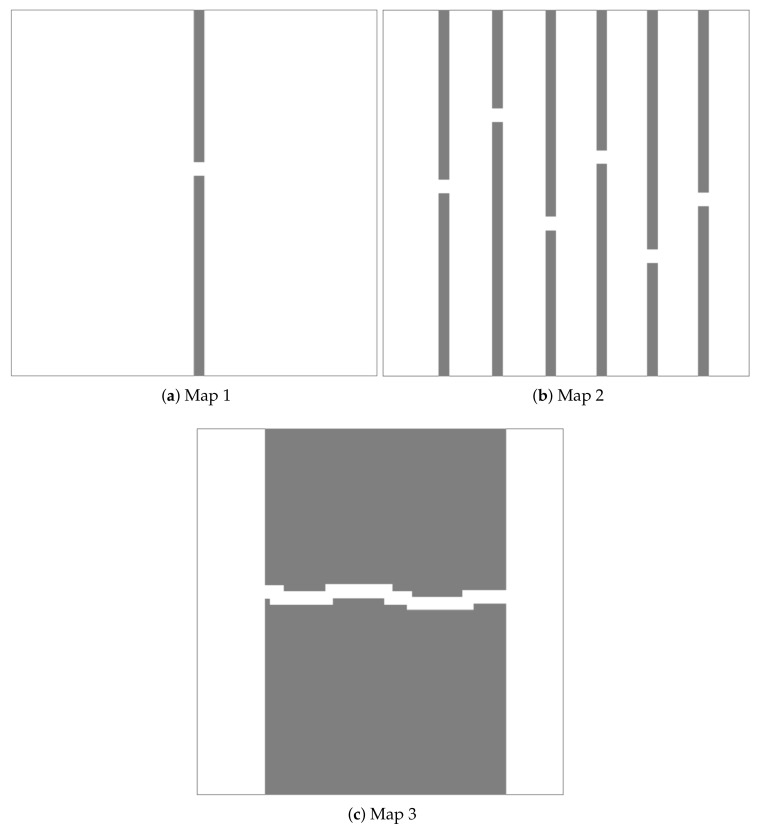
Three different maps with narrow passages. Map 2 has five more narrow passages than Map 1, and the narrow passage in Map 3 is longer than that in Map 2.

In the first part, three different maps were created with the size 800 × 800 (Figure 8). The start and goal point coordinates were set to (50,50) and (750,750), respectively. The step size of BS-RRT and narrow passage width were set to 30. The global path was generated in a map with narrow passages by the BS-RRT algorithm. Then, the cyclic pruning algorithm was used to optimize the generated global path. We validate the performance of BS-RRT algorithm and cyclic pruning algorithm by comparing BS-RRT with RRT, RRT-Connect, P-RRT, 1-0 Bg-RRT [28], and RRT${}^{*}$. We set P=0.5 in P-RRT. For the RRT${}^{*}$ algorithm, the earliest time to finish the feasible path planning was set as the criterion.

In the second part, we predicted the trajectories of dynamic flight obstacles by using RMGM(1,1) models to prepare for local path adjustments. The accuracy of the RMGM(1,1) model was compared with other models. First, the ECGM(1,1) model of exponential curve optimization, PCGM(1,1) model of polynomial curve optimization in [39], and original GM(1,1) model were compared with RMGM(1,1). Second, the metabolic MGM(1,1) and GDF [47] were compared with RMGM(1,1). Third, the local path adjustment scheme was implemented according to the prediction results of the RMGM(1,1) model.

In this section, the dynamic flight obstacle was set as the same model as the UAV, and the radius of the envelope ball is 0.5. Considering the unforeseeable factors in the actual UAV flight and the errors caused by the prediction model, the setting of the *R* value was generally greater than the sum of the radius of the envelope circle between the UAV and the dynamic obstacle, and *R* was set to 2. Some of the necessary parameters are summarized in Table 1.

### 4.1. BS-RRT and the Circular Pruning Algorithm

**Figure 9:**
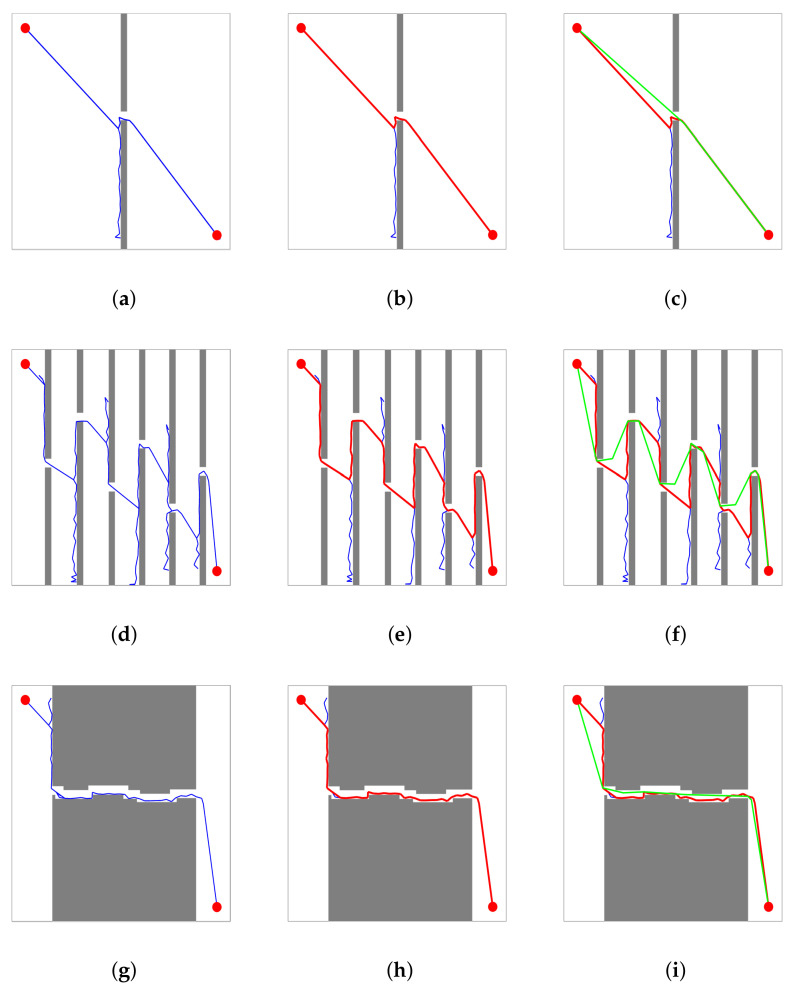
Planning results of BS-RRT and cyclic pruning algorithms in the three maps. The blue line refers to the path of the BS-RRT algorithm, the red line represents the backtracking path after BS-RRT connects the starting point and the end point, and the green line corresponds to the final path after cyclic pruning. (**a**,**d**,**g**) show the running results of BS-RRT algorithm in three maps; (**b**,**e**,**h**) show the backtracking path of BS-RRT algorithm in three maps; (**c**,**f**,**i**) show the final path generated after pruning in three maps.

More detailed data on Figure 9 are documented in Table 2. The narrow passages in Figure 9c are few and short. The path generated by the BS-RRT algorithm was close to the optimal path before pruning, and a path length reduction of 5.606% was achieved after pruning by using the cyclic pruning algorithm. As shown in Figure 9f,i, the number and length of narrow channels increased, and the path generated by the BS-RRT algorithm was more tortuous and different from the optimal path. The effect of the cyclic pruning algorithm is obvious, and the path reductions were 24.898% and 17.077%.

**Figure 10:**
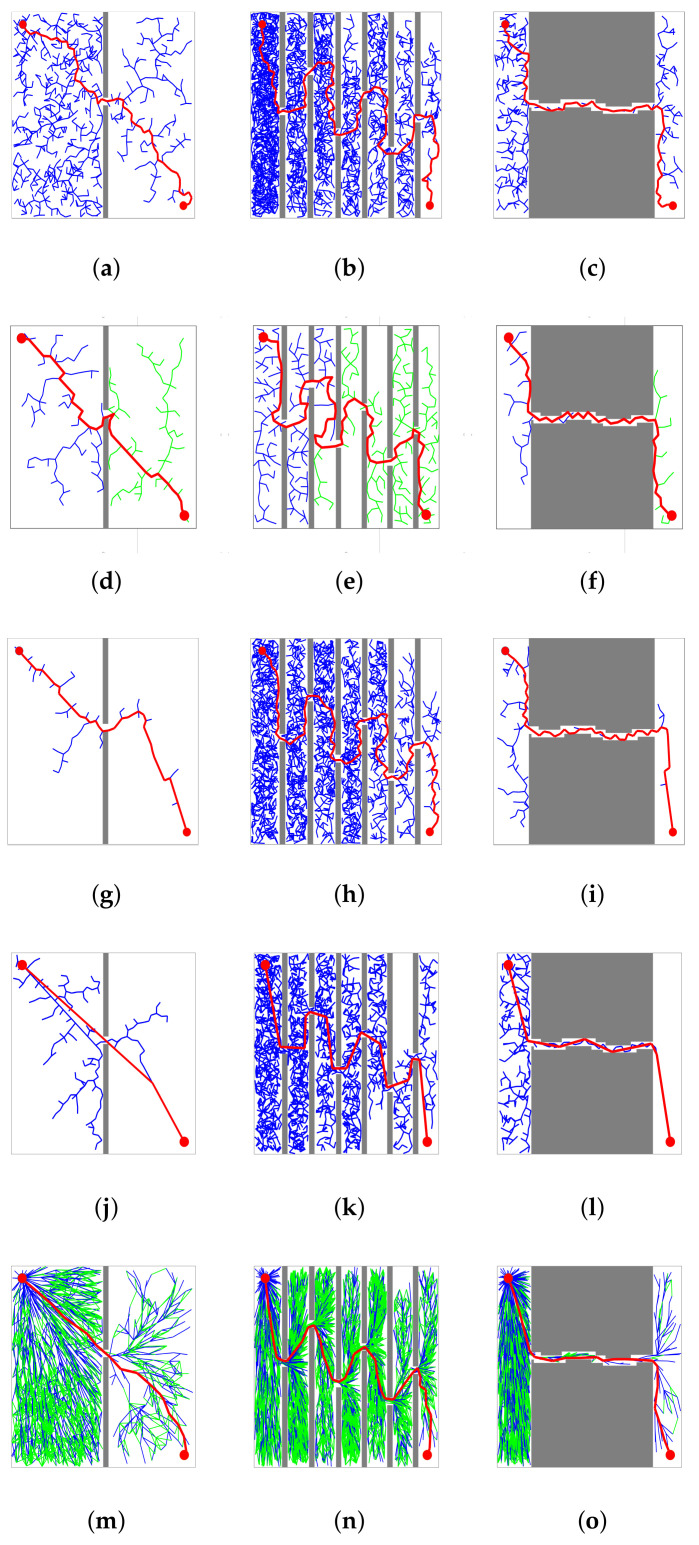
(**a**–**c**) show the path planning results of RRT in three maps; (**d**–**f**) are the path planning results of RRT-Connect in three maps; (**g**–**i**) are the path planning results of P-RRT in three maps; (**j**–**l**) are the path planning results of 1-0 Bg-RRT in three maps; and (**m**–**o**) are the path planning results of RRT${}^{*}$ in three maps. The red line corresponds to the final path.

In Figure 10, RRT, RRT-Connect, P-RRT, 1-0 Bg-RRT, and RRT${}^{*}$ carry out global path planning in Maps 1, 2, and 3. The final backtracking paths are relatively tortuous when the number of narrow passages increases.

**Figure 11:**
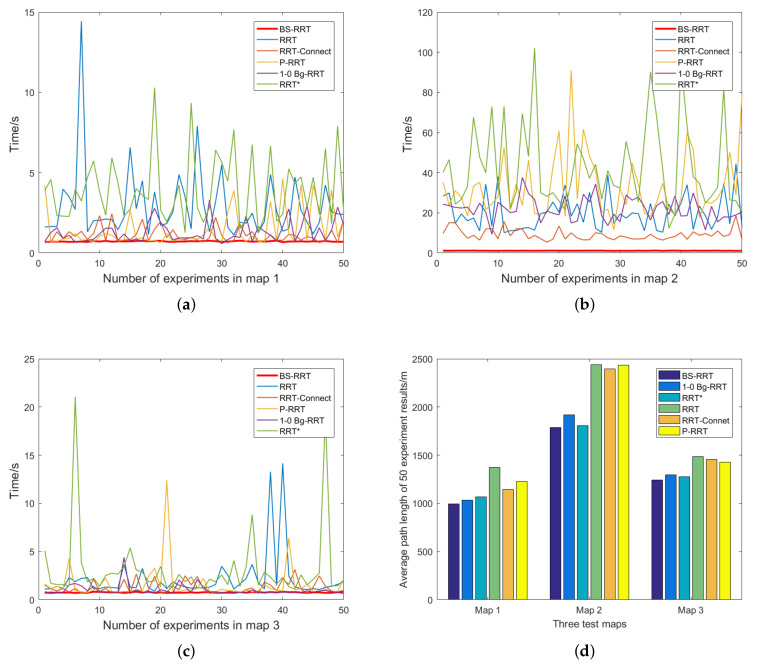
(**a**–**c**) show the run time for each of the 50 experiments for BS-RRT, RRT, RRT-Connect, P-RRT, 1-0 Bg-RRT, and RRT${}^{*}$ in the three maps; (**d**) shows the average path length of 50 experiments using the six algorithms.

In Figure 11a–c, each run time is recorded for BS-RRT, RRT, RRT-Connect, P-RRT, 1-0 Bg-RRT, and RRT${}^{*}$ of path planning in Maps 1, 2, and 3 in 50 experiments. Intuitively, except for BS-RRT, the stability of the other algorithms is poor, and the time fluctuates greatly with respect to the path planning of each experiment. Moreover, as shown in Figure 11b, when the narrow passages increase, the path planning time of the five algorithms extends significantly, except for BS-RRT, and the fluctuation range of the algorithm planning time is larger than that of BS-RRT.

In Figure 11d, the average path length of 50 times the path planning of the six algorithms is recorded. The paths planned by the BS-RRT algorithm are all optimal. Table 3 is drawn based on computer simulation results. According to Table 3, in terms of the average planning time, the BS-RRT algorithm had the fastest convergence rate. In the three maps, the convergence times were 0.722, 1.159, and 0.757 s. With the increase in the number of narrow passages, the convergence time of BS-RRT rises slightly, and the length of the narrow passages does not affect the convergence time of the algorithm. The convergence time of the other algorithms varies greatly with the increase in the number of narrow passages.

In Map 2, the mean convergence times of RRT, RRT-Connect, P-RRT, 1-0 Bg-RRT, and RRT${}^{*}$ were 20.02, 9.152, 33.216, 21.898, and 42.886 s. In terms of the maximum time difference of the algorithm, the time fluctuations of BS-RRT algorithm were the least among the three maps, which were 0.097, 0.162, and 0.136 s. Similarly, the volatility of other algorithms increased with the increase in the number of narrow passages. The maximum fluctuation times of the path planning with other algorithms in Map 2 were 34.189, 18.563, 91.061, 28.304, and 89.655s. Although the 1-0 Bg-RRT planning path was more efficient in the state space with a narrow passage, the efficiency of the algorithm decreased with the increase of the number of narrow passages. In terms of the average planned path length, the path was optimized by the circular pruning algorithm to be the shortest in the three maps.

**Figure 12:**
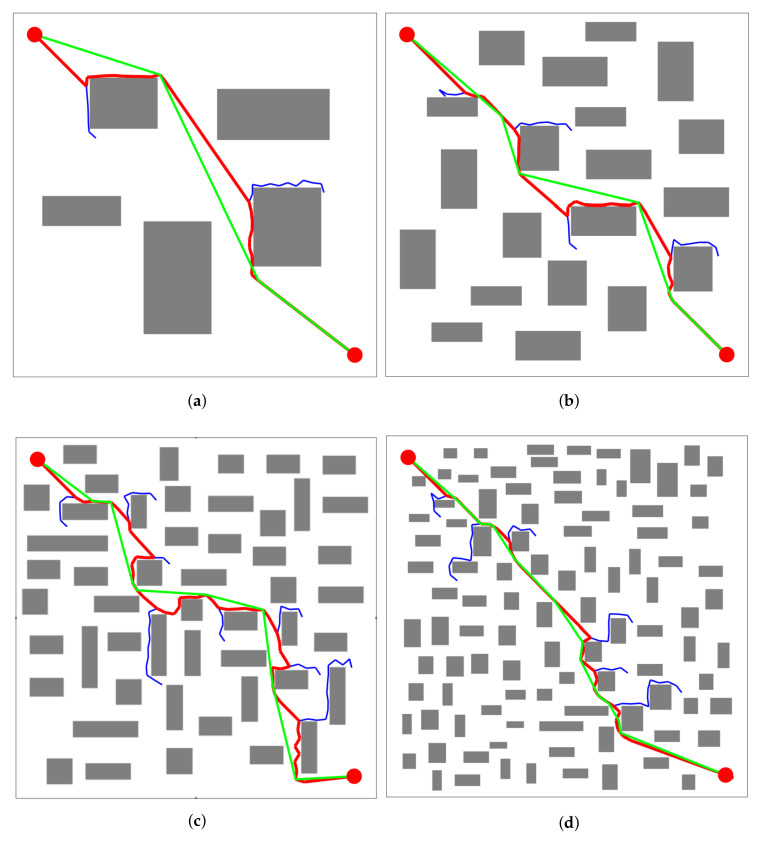
Path planning of the BS-RRT algorithm in common maps and there are 5, 20, 50 and 100 obstacles in (**a**–**d**) respectively.

Table 4 records the time taken for BS-RRT to plan 50 times in the four maps shown in Figure 12. The convergence time and fluctuation of the algorithm slightly increased when the number of obstacles gradually rose, and the maximum fluctuation was 0.2754 s. The average convergence times of the algorithm in the four maps were 0.8169, 0.8556, 0.9452, and 0.9456 s. These results indicate that, in ordinary maps, BS-RRT also maintains good convergence speed and stability.

Table 5 records the detailed data of the path changes before and after pruning using the circular pruning algorithm in the four maps in Figure 12. The path length and the tortuous degree generated by BS-RRT increased with the number of obstacles. In Figure 12c, the zigzag degree of the path was the largest when the obstacle was 50, and the pruning effect, which was reduced by 11.46%, was the most obvious. As shown in Figure 12a, the path generated by BS-RRT was close to the optimal path with a low tortuous degree, and the path length was reduced by 5.33% after pruning. In Figure 12b,d, the path tortuous degree is similar in the two figures, and the path length decreased by 9.37% and 9.75%, respectively.

### 4.2. RMGM(1,1) Model Accuracy Comparison

To intuitively express the accuracy of the model, we define the following formula. Three axes of coordinates are split to build the original data sequence

(20) $$P^{\left(0\right)}\left(k\right)=\left(X^{\left(0\right)}\left(k\right),Y^{\left(0\right)}\left(k\right),Z^{\left(0\right)}\left(k\right)\right).$$

where

(21) $$\left\{\begin{array}{c} \mathrm{Axis}x:X^{\left(0\right)}=\left\{x^{\left(0\right)}\left(k\right)\right\}\\ \mathrm{Axis}y:Y^{\left(0\right)}=\left\{y^{\left(0\right)}\left(k\right)\right\}\\ \mathrm{Axis}z:Z^{\left(0\right)}=\left\{z^{\left(0\right)}\left(k\right)\right\} \end{array}\right.,k=1,2,\ldots ,n.$$

The coordinates obtained by the prediction of the three coordinate sequences are the fitting values

(22) $$\hat{P}\left(k+t\right)=\left({\hat{X}}^{\left(0\right)}\left(k+t\right),{\hat{Y}}^{\left(0\right)}\left(k+t\right),{\hat{Z}}^{\left(0\right)}\left(k+t\right)\right).$$

where

(23) $$\left\{\begin{array}{c} \mathrm{Axis}x:{\hat{X}}^{\left(0\right)}=\left\{{\hat{x}}^{\left(0\right)}\left(k+t\right)\right\}\\ \mathrm{Axis}y:{\hat{Y}}^{\left(0\right)}=\left\{{\hat{y}}^{\left(0\right)}\left(k+t\right)\right\}\\ \mathrm{Axis}z:{\hat{Z}}^{\left(0\right)}=\left\{{\hat{z}}^{\left(0\right)}\left(k+t\right)\right\} \end{array}\right.,t=1,2,\ldots ,m.$$

To evaluate the accuracy of the prediction effect of the model, we define the compound position error as

(24) $$E_{p}=\sqrt{E_{x}^{2}+E_{y}^{2}+E_{z}^{2}}.$$

$E_{x},E_{y},E_{z}$ are the position errors in the three directions and are defined as follows

(25) $$\left\{\begin{array}{c} E_{x}=\left|\hat{X}-X\right|\\ E_{y}=\left|\hat{Y}-Y\right|\\ E_{z}=\left|\hat{Z}-Z\right| \end{array}.\right.$$

$\hat{X},\hat{Y},$ and $\hat{Z}$ are the coordinate components of the three directions calculated by the model and are the three directions of the corresponding real coordinates for the moving target

(26) $$\left\{\begin{array}{c} E_{\max}=\max_{1}^{N}\left(E_{p}\right)\\ E_{\min}=\min_{1}^{N}\left(E_{p}\right)\\ E_{\mathrm{eq}}=\sum_{1}^{N}\left(E_{p}\right) \end{array}\right..$$

**Figure 13:**
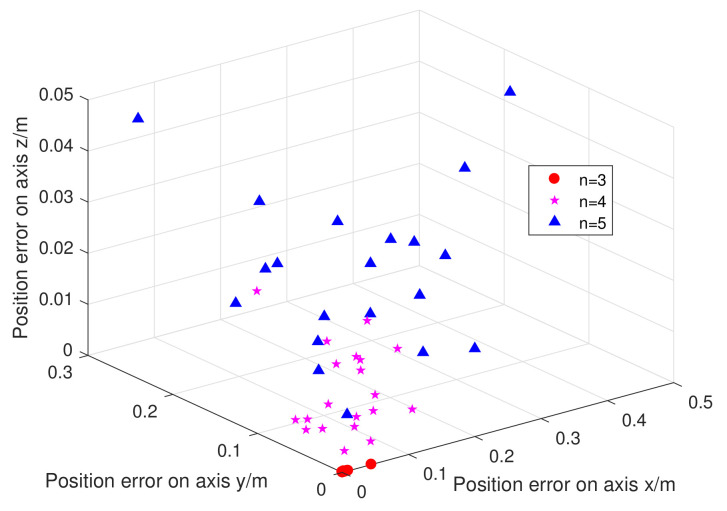
The error between the predicted and the actual coordinates at n = 3, 4, and 5.

The model length *n* determines the metabolic rate and accuracy of the model. Thus, discussing the model length *n* is necessary. According to [48,49], the length of the model is usually between 3 and 5. In Figure 13, we predict 20 sets of coordinates and calculate the error by comparing them with the real coordinates. The red dot is closest to the origin, and the prediction error is the smallest when the model length is 3. Therefore, we chose the model length of *n* = 3 as the optimal parameter value.

**Figure 14:**
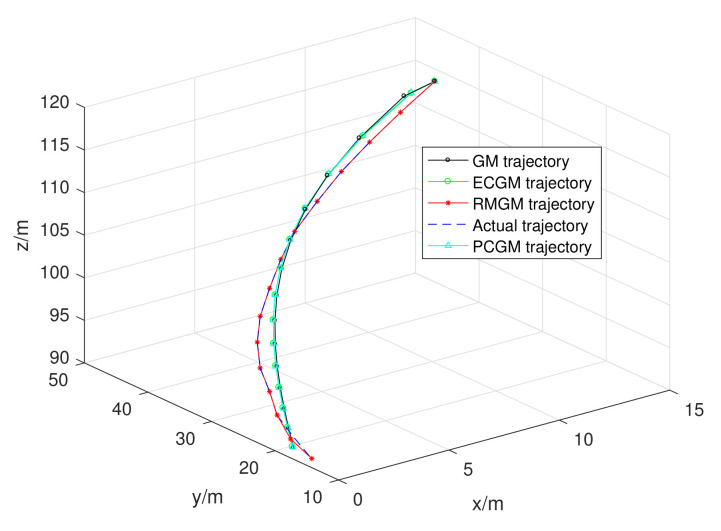
Predicted trajectories of the GM, ECGM, PCGM, and RMGM models.

**Figure 15:**
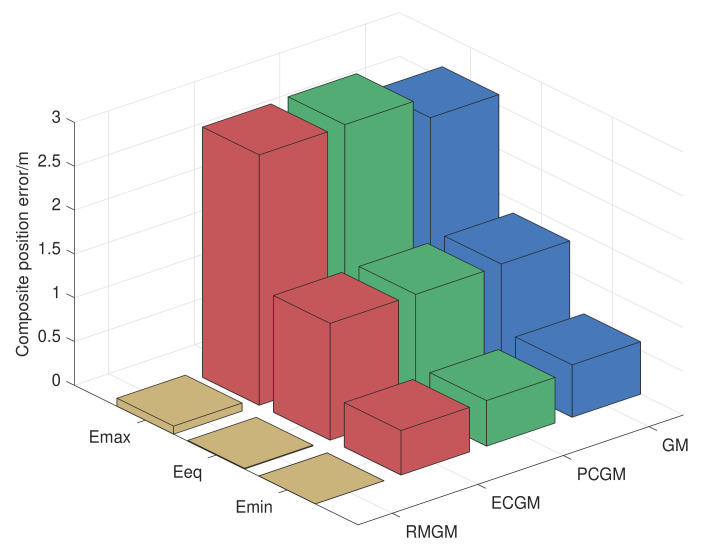
Composite position error calculated by the GM, ECGM, PCGM, and RMGM models in the experiment.

The X axis in Figure 14 shows the real-time position of the moving object in the X direction. Similarly, the Y and Z axes represent the real-time position of the moving object in the Y and Z directions, respectively. The trajectories predicted by RMGM, ECGM, PCGM, and GM, as well as the actual trajectories of moving objects, are shown in Figure 14. Except for the red curve that represents RMGM, the other curves have large deviations from the real trajectory. To show the trajectory prediction accuracy of the different methods clearly, Figure 15 depicts the combined position errors calculated by RMGM, ECGM, PCGM, and GM.

Yellow, red, green, and blue bars represent the RMGM, ECGM, PCGM, and GM errors, respectively. The errors of RMGM are far less than those of the other models. Table 6 is drawn according to the computer simulation results, According to Table 6, the composite position errors calculated by RMGM were much smaller than those of the other models. The average errors of ECGM, PCGM, and GM were 120.17-, 120.32-, and 121.38-times that of RMGM, respectively.

**Figure 16:**
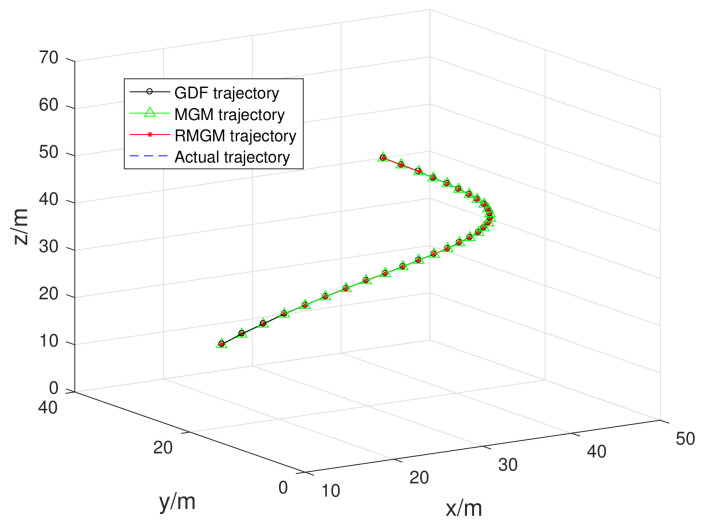
Trajectory prediction of the GDF, MGM, and RMGM models.

**Figure 17:**
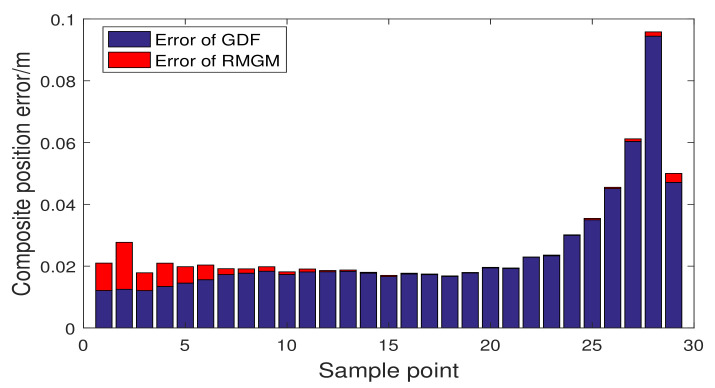
Combined position errors of GDF and RMGM.

**Figure 18:**
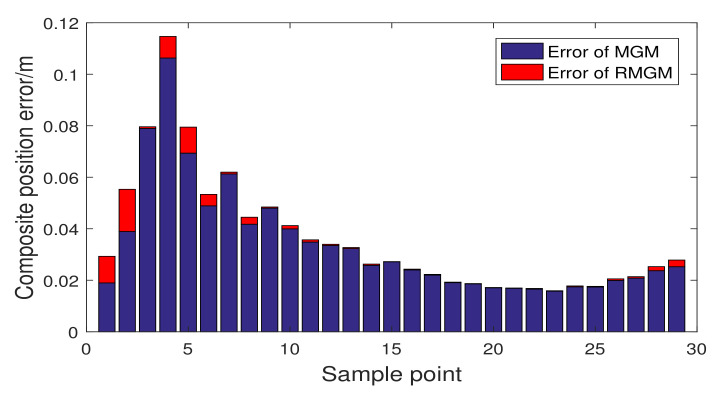
Combined position errors of MGM and RMGM.

In Figure 16, we compare the trajectory prediction performance of RMGM, GDF, and MGM models, and the prediction trajectories of the three models are close to the real trajectory. Distinguishing the advantages from the disadvantages in the trajectory diagram is difficult. In Figure 17 and Figure 18, the fitting position errors of the three models can be shown clearly. Furthermore, the errors of RMGM are the minimum. In Figure 17, the variation trend of the RMGM error is opposite to that of GDF. The error of the GDF model increased when the background value error was small because the GDF used an error correction term to reduce the model error.

**Figure 19:**
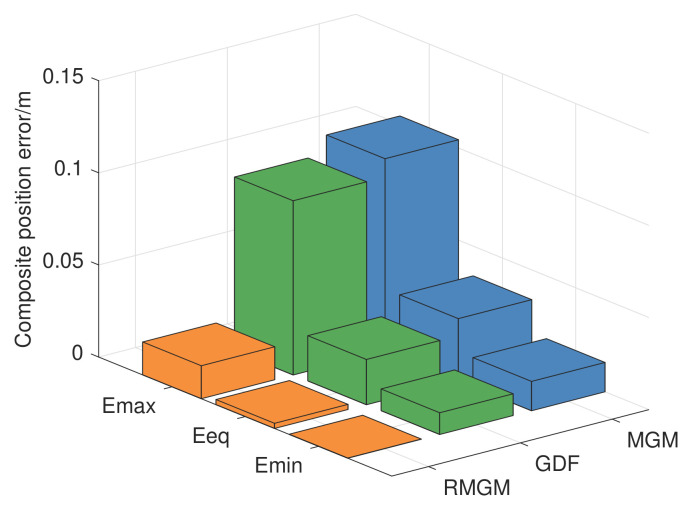
Comparison of the prediction errors of GDF, MGM, and RMGM.

Moreover, given that the error correction term is a prediction model, the variation trend of the GDF model’s error was hysteretic compared with the RMGM. In Figure 18, considering that the RMGM model only optimized the background value error in the modeling process, the error followed the same trend as that of the MGM model. Figure 19 and Table 7 show a comparison of the compound position errors calculated by the three methods. The average synthetic position errors of MGM, GDF, and RMGM were 0.03437, 0.02435, and 0.00273, respectively. The average position errors of MGM and GDF models were 12.59 times and 8.92 times the RMGM models, respectively. Clearly, RMGM had better trajectory prediction performance than MGM and GDF.

### 4.3. Local Path Adjustment

**Figure 20:**
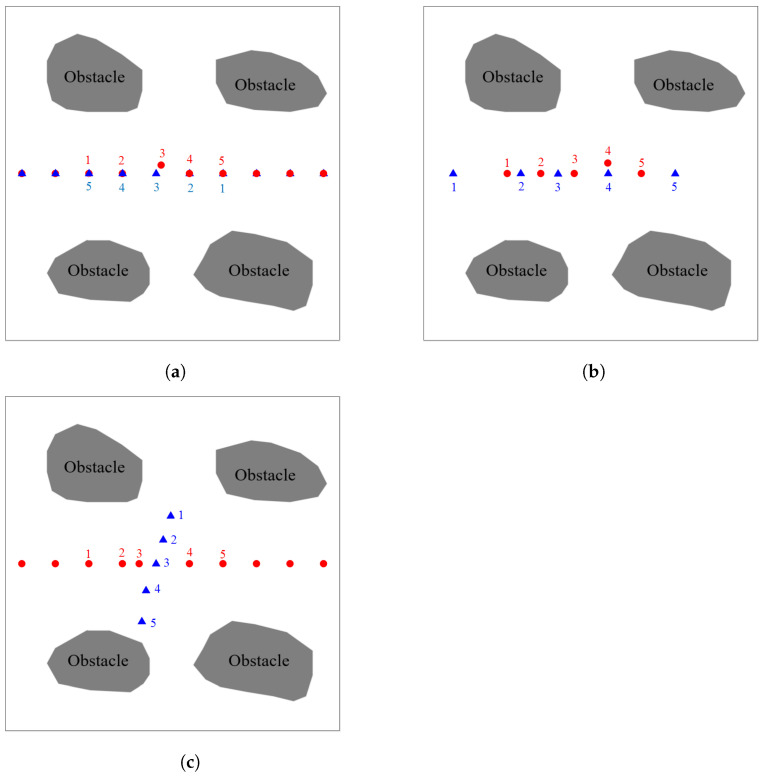
Dynamic flight obstacles are represented as blue triangles, the red circle is the UAV, and the number of the corresponding color is the coordinate point of the corresponding time. (**a**,**b**) show the local path adjustment for the opposite and codirectional obstacles, respectively. (**c**) shows the deceleration strategy of the UAV.

In Figure 20, The local path adjustment strategy is demonstrated on an 80 × 80 size map. In Figure 20c, the local path adjustment of the same direction obstacle and the opposite obstacle are classified into one category because they have the same deceleration strategy, and the local adjustment of the opposite obstacle is taken as an example.

Table 8 records the coordinate information of the UAV and the dynamic flying obstacle in Figure 20a at every moment. The coordinate of the next moment about the dynamic flying obstacle was recorded by using the RMGM(1,1) model. R1 refers to the distance between the predicted coordinates of the dynamic flying obstacle and the UAV in the next moment. R2 is the distance between the dynamic flying obstacle and UAV after the UAV adopts the deceleration strategy in the next moment.

The adjusted coordinates indicated that the original coordinates at the next moment are replaced by the adjusted coordinates. R3 refers to the distance between the coordinates of the dynamic flying obstacle and coordinates after the UAV adopts a change strategy. The safe distance of R1, R2, and *R* was 2. According to the prediction results, R1 was 1.2 when the UAV flies to the coordinates at 2 s, that is, collision occurred between the UAV and the dynamic flying obstacle at 3 s.

First, a deceleration strategy was validated. R2 was 0 if the UAV adopted the deceleration strategy, suggesting that the path of the UAV coincided with the trajectory of the dynamic obstacle. A decentralized strategy was adopted instead of the deceleration strategy. The distance between the offset coordinates of the UAV and the predicted coordinates of the dynamic flight obstacle was greater than 2. Thus, the coordinate (36, 40) in the path was replaced by the coordinate (37.2, 38). R3 was 2.3 at 3 s, and the obstacle avoidance was successful.

The coordinate information of the UAV and dynamic flight obstacle in Figure 20b at each moment is recorded in Table 9. R1 was 0.2 at 3 s, suggesting that a collision between the UAV and dynamic flight obstacle occur at 4 s. First, the feasibility of a deceleration strategy was verified. R2 was 0 if the UAV adopted the deceleration strategy. Thus, a decentralized strategy was adopted, and the coordinates (43.8, 38) were added to the path of the UAV instead of (44, 40) at 4 s. R3 was 2.3 when the UAV flew to the coordinates (43.8, 38) at 4 s, and the obstacle avoidance was successful.

The coordinate information of teh UAV and dynamic flight obstacle in Figure 20c at each moment is recorded in Table 10. Considering that the trajectories of UAVs and dynamic flying obstacles are not coincident, the UAV only needs to avoid dynamic flight obstacles by adopting a deceleration strategy. Oncoming obstacles are used as an example. Collision occureed at 3 s when R1 was 1. The feasibility of the deceleration strategy was verified. R2 was 4.02 at 3 s when the deceleration strategy was carried out. Hence, *t* no collisions occurred at 3 s. Then, the coordinates were added into the path after the deceleration strategy, and the obstacle avoidance was successful.

## 5. Conclusions

In this paper, we presented an autonomous flight scheme of UAVs in complex urban environments. This scheme is a composite method that includes global path generation and local path adjustment. In the global path planning, we presented a branching selection RRT(BS-RRT) algorithm to plan the path in urban environment with many narrow passages. The experimental results showed that BS-RRT could plan global paths quickly by branching selection continually. BS-RRT converged faster in narrow passage environments compared with RRT, RRT-Connect, P-RRT, 1-0 Bg-RRT, and RRT${}^{*}$.

The BS-RRT still maintained a fast convergence speed and high stability in the ordinary map with different numbers of obstacles after many experiments. We also proposed a cyclic pruning algorithm to optimize the path generated by BS-RRT. The simulation results show that the cyclic pruning algorithm shortened the path, and the path after pruning could reach the optimal path. The effect of the cyclic pruning algorithm was enhanced with the increase in the tortuous degree of the path. Forecasting and decision making were included in the local path adjustment scheme, and the RMGM(1,1) model was proposed to predict the trajectory of the dynamic flight obstacles.

The real-time performance of the RMGM(1,1) model was guaranteed by constantly adding new coordinates and eliminating old coordinates. The optimal model length was selected to improve the prediction accuracy. Then, RMGM(1,1) was compared with the other trajectory prediction methods (e.g., GM(1,1), ECGM(1,1), PCGM(1,1), MGM(1,1), and GDF) in the computer simulation. The simulation results show that the trajectory prediction performance of RMGM was superior to the other models. The decision of the UAV was based on the prediction results of the RMGM model. The decision section contained two path adjustment strategies.

If the coordinate position after deceleration was far from the trajectory of the dynamic flight obstacle, then the deceleration strategy was feasible. The use of the deceleration strategy was preferred when the trajectory of the dynamic flight obstacle did not coincide with the path of the UAV. The decentralized strategy was used when the trajectory of the dynamic flight obstacle coincided with the path of the UAV. The results of the prediction model are the premise for both kinds of decision making. The experimental results demonstrated the effectiveness of the local path adjustment scheme.

## Figures and Tables

**Table 1:** The main parameters in the experiment.

Experiment	Parameters
Runtime environment	IntelCPU2.8G 16 GB memory computer using MATLAB-R2016B
Map size	800 × 800
Number of maps	3
Start point in the map	(50,50)
Goal point in the map	(750,750)
Narrow passage width	30
Envelope sphere radius of UA V and dynamic flight obstacle	0.5
Narrow passage width	30
Distance R	2

**Table 2:** Comparison of path changes before and after pruning with cyclic pruning algorithms.

Figure	Before Pruning	After Pruning	Percentage Reduction
Figure 9c	1054.345	995.238	5.606%
Figure 9f	2369.049	1779.210	24.898%
Figure 9i	1505.181	1248.134	17.077%

**Table 3:** Time data from 50 experiments for the six algorithms.

Map	Algorithm	Average Planning Time/s	Maximum Time Difference/s	Average Path Length
Map 1	BS-RRT	0.722	0.097	994.858
	RRT	2.905	13.324	1374.577
	RRT-Connect	1.121	2.449	1146.323
	P-RRT	1.466	4.621	1225.728
	1-0 Bg-RRT	1.304	2.628	1033.617
	RRT${}^{*}$	3.951	9.050	1068.216
Map 2	BS-RRT	1.159	0.162	1787.360
	RRT	20.020	34.189	2439.182
	RRT-Connect	9.152	18.563	2396.566
	P-RRT	33.216	91.061	2433.546
	1-0 Bg-RRT	21.898	28.304	1920.761
	RRT${}^{*}$	42.886	89.655	1807.956
Map 3	BS-RRT	0.757	0.136	1242.084
	RRT	2.127	13.274	1486.449
	RRT-Connect	1.270	3.126	1457.493
	P-RRT	1.567	12.392	1429.350
	1-0 Bg-RRT	1.008	3.768	1298.581
	RRT${}^{*}$	3.115	19.911	1274.754

**Table 4:** Time data from 50 experiments for BS-RRT algorithms in common maps.

Figure	Maximum Time Difference/s	Minimum Planning Time/s	Average Planning Time/s	Maximum Planning Time/s
Figure 12a	0.1277	0.7388	0.8169	0.8665
Figure 12b	0.1367	0.7632	0.8556	0.8999
Figure 12c	0.1530	0.8429	0.9452	0.9959
Figure 12d	0.2754	0.8387	0.9456	1.1141

**Table 5:** Comparison of path changes before and after pruning with cyclic pruning algorithms.

Figure	Before Pruning	After Pruning	Percentage Reduction
Figure 12a	1105.35	1046.38	5.33%
Figure 12b	1168.98	1059.44	9.37%
Figure 12c	1199.60	1062.18	11.46%
Figure 12d	1228.31	1108.53	9.75%

**Table 6:** Composite position error.

Method	Emin	Eeq	Emax
RMGM	0.0002	0.0112	0.1238
ECGM	0.4897	1.3459	2.6561
PCGM	0.4985	1.3476	2.6816
GM	0.5676	1.3595	2.5258

**Table 7:** Comparison of the composite position error calculated by three methods.

Method	Emin	Eeq	Emax
MGM	0.01531	0.03437	0.11419
GDF	0.01163	0.02435	0.09092
RMGM	0.00005	0.00273	0.01587

**Table 8:** Coordinate information of the local path adjustment in Figure 20a.

Time	UAV Coordinates	Obstacle Coordinates	Prediction Coordinates	R1	R2	Adjusted Coordinate	R3
1 s	(20,40)	(52,40)	(45,40)	17			
2 s	(28,40)	(44,40)	(37.2,40)	1.2	0	(37.2,38)	2.3
3 s	(36,40)	(36,40)	(29.4,40)	14.6			
4 s	(44,40)	(28,40)	(21.8,40)	30.2			
5 s	(52,40)	(20,40)	(14.3,40)				

**Table 9:** Coordinate information of the local path adjustment in Figure 20b.

Time	UAV Coordinates	Obstacle Coordinates	Prediction Coordinates	R1	R2	Adjusted Coordinate	R3
1 s	(20,40)	(7,40)	(23.6,40)	4.4			
2 s	(28,40)	(23,40)	(32.2,40)	3.8			
3 s	(36,40)	(32,40)	(43.8,40)	0.2	0	(43.8,38)	2.3
4 s	(44,40)	(44,40)	(59.2,40)	7.2			
5 s	(52,40)	(60,40)	(112,40)				

**Table 10:** Coordinate information of the local path adjustment in Figure 20c.

Time	UAV Coordinates	Obstacle Coordinates	Prediction Coordinates	R1	R2	Adjusted Coordinate	R3
1 s	(20,40)	(39.5,28.6)	(37.7,34.3)	11.25			
2 s	(28,40)	(37.7,34.3)	(35.9,41)	1	4.02	(32,40)	
3 s	(36,40)	(36,40)	(34.3,46.6)	11.73			
4 s	(44,40)	(33.6,46.4)	(31.4,53.8)	24.80			
5 s	(52,40)	(32.6,53.8)	(31.6,62.3)				

## Data Availability

Not applicable.
